# Enteric glia: extent, cohesion, axonal contacts, membrane separations and mitochondria in Auerbach’s ganglia of guinea pigs

**DOI:** 10.1007/s00441-022-03656-3

**Published:** 2022-06-22

**Authors:** Giorgio Gabella

**Affiliations:** University College London, London, WC1E 6BT UK

**Keywords:** Enteric glia, Myenteric plexus, Auerbach’s ganglia, Glial cells, Cell adhesion

## Abstract

Studied by electron microscopy and morphometry, Auerbach’s ganglia comprise nerve cell bodies that occupy ~ 40% of volume; of the neuropil, little over 30% is neural processes (axons, dendrites) and little less than 30% is glia (cell bodies, processes). The amount of surface membrane of neural elements only marginally exceeds that of glia. Glial cells extend laminar processes radially between axons, reaching the ganglion’s surface with specialized membrane domains. Nerve cells and glia are tightly associated, eliminating any free space in ganglia. Glia expands maximally its cell membrane with a minimum of cytoplasm, contacting a maximal number of axons, which, with their near-circular profile, have minimal surface for a given volume. Shape of glia is moulded by the neural elements (predominantly concave the first, predominantly convex the second); the glia extends its processes to maximize contact with neural elements. Yet, a majority of axons is not reached by glia and only few are wrapped by it. Despite the large number of cells, the glia is not sufficiently developed to wrap around or just contact many of the neural elements. Mitochondria are markedly fewer in glia than in neurons, indicating a lower metabolic rate. Compactness of ganglia, their near-circular profile, absence of spaces between elements and ability to withstand extensive deformation suggest strong adhesion between the cellular elements, holding them together and keeping them at a fixed distance. Many axonal varicosities, with vesicles and membrane densities, abut on non-specialized areas of glia, suggesting the possibility of neurotransmitters being released outside synaptic sites.

## Introduction

An essential feature of nervous tissue is a close association, both structural and functional, between two cell populations, neurons and glia, some kind of partnership. In situ, the two components almost invariably occur together. This association is also evident in autonomic ganglia, and it presents some special aspects in the enteric ganglia. Enteric neurons and enteric glia appear inseparable: Glial cells and processes are never found that are not associated with a neural structure.

A large population of glial cells is part of the enteric ganglia, especially the Auerbach’s ganglia and its interconnecting strands (and to a lesser extent the Meissner’s or submucosal ganglia). At the time of the first observations, the term “enteric glia” was used, and the cells were seen as specific of the ganglia and the connecting strands. Glial cells that accompany nerve bundles in the musculature and in the mucosa were regarded as standard equivalents of the glial cells of all autonomic nerves. The early studies documented, by light and electron microscopy, the fine structural features of the enteric glia, as it was then envisaged (Gabella 1971, 1972, 1981; Cook and Burnstock 1976; Komuro et al. 1982). Some immunochemical markers of these cells were quickly identified (Jessen and Mirsky 1980; Ferri et al. 1982; Björklund et al. 1984); intracellular injections of a tracer (HRP) outlined the full extension of these cells (Hanani and Reichenbach 1994). Chemical markers, such as glial fibrillary acidic protein (GFAP) and S100b, have become key tools to investigate these cells in vivo (Hoff et al. 2008) and, far more frequently, in vitro; the transcription factor Sox8/9/10, located in the nucleus, has also been useful for cell counts (Hoff et al. 2008). There are comprehensive review articles on the early studies (e.g. Gershon and Rothman 1991; Rühl 2005) and a full historical essay (Gulbransen 2014) is available.

In the last 12 years or so a renewed interest in the enteric glia has vastly extended the number and locations of the enteric glial cells, and has produced a large literature, including comprehensive review articles (Coelho-Aguiar et al. 2015; Grubisic and Gulbransen 2017). There are tentative identifications of several “types” of enteric glial cells (of which the intraganglionic ones examined here are only a small part). When four morphologically distinct populations (or “types”) are recognized, the glial cells of the ganglia are the first type. The other types include extraganglionic glial cell, those accompanying the intramuscular nerves (nerve bundles, in the nomenclature adopted here) and those found in the mucosa (Gulbransen and Sharkey 2012; Boesman et al. 2015).

The roles attributed to the various glial cells are numerous, wide and essential for the functions of the intestine, especially regarding the cells found in the mucosa. In general terms, the modern studies agree that enteric glial cells play a major role in “intestinal homeostasis”; indeed, they are regarded as being at centre stage in the communication between the many cellular elements of the intestine. Those of the mucosa in particular (“inflammatory enteric glial cells”) appear to have a major immunological role, hence, to be essential to the intestinal epithelial barrier (De Giorgio et al. 2012; Pochard et al. 2018). These are the cells that have attracted a wide interest, strengthened by a possible significance in human intestinal diseases (Gulbransen and Sharkey 2012; Sharkey 2015; Pochard et al. 2018). Also, there is convergence with the modern perspectives on the glia of the CNS, the importance of which is encapsulated in their definition as “non-neuronal cells in the central (CNS) and peripheral nervous system (PNS) that nourish neurons and maintain homeostasis” (Grubisic and Gulbransen 2017). Even within the Auerbach’s plexus several types or subtypes of glial cells have been described in good detail and have refined the cell assignments of the early histochemical observations (Boesman et al. 2015). The source of the heterogeneity supported also by electrophysiological studies (Hanani et al. 1989)—whether genetic and developmental or functional and adaptive—remains an open question (Boemans et al. 2015), and these authors wisely stress the importance of cell plasticity and the different expression of chemical markers within the cell populations.

The new data presented here are by electron microscopy and deal exclusively with the Auerbach’s ganglia, which were examined in situ, in adult guinea pigs. The observations and the discussion do not contribute to our knowledge of the glial cells described in the literature in other parts of the intestinal wall. Most of the current research aims to discover molecular and functional features of these cells that may assist the activity of the organ—and are of benefit to the individual on top of it. An additional aim, adopted here, holding a further-away and less-practical objective, is a better understanding of morphogenesis, that is, of the set of processes (mechanical, molecular, electrical and more) that brings about the local features. Some features coming out of morphogenesis (and changes and innovations) have been preserved in evolution; others must have been discarded, again in evolution, that is on the basis of the advantage, or the disadvantage, imparted to an individual (as a member of a large population), and many more probably have no positive or negative survival value. Studies of morphogenesis, such as the present one, look at these features at their own level, rather than at the level of the organism. Large assemblies of many different elements, in a living tissue, such as one finds in these microscopic ganglia, prompt the question of how that assembly was formed, how it is maintained, especially if there is some order and some consistency or regularity between the individuals.

## Material and methods

### Materials

The observations are all from Auerbach’s ganglia (myenteric ganglia) and connecting strands of the ileum of guinea pigs. For preliminary observations, several scores of animals were used, male and female. No differences in ultrastructure were noted between males and females, and only modest differences from birth to old age. For the quantitative work, the material from about 20 guinea pigs was gathered. The selected animals were close in age and were of either sex. The seven ganglia documented in the tables were from six different animals.

Comparative observations on other ganglia of the gut and on other species are ignored here. All the procedures involving materials from animals complied fully with the UK Home Office Regulations under a Personal and a Project License to the Author. The material was obtained from adult guinea pigs (200–600 g in body weight, of either sex), shortly after they had been killed with an overdose of anaesthetic (Nembutal). Loops of the small intestine from the ileal region, a few centimetre long, were extracted. Distended by intraluminar injection of oxygenated Krebs solution, between ligatures, the loops were immersed in fixative at room temperature, for about 1 h. Some loops were left to empty and then stimulated (with 10^−6^ M carbachol) to contract, unimpaired or after expanding either the circumference or the length, and then immersed in fixative. In all cases, the intestinal loops were then cut into short rings (3–5 mm), kept immersed in fixative for another 2–18 h and then processed and embedded in resin without further trimming. In these relatively large specimens, orientation and position of the ganglia in the intestinal wall remained always known, even at the time of section cutting in much trimmed down resin blocks.

### Histology

The most consistent results were obtained with a fixative made of 5% glutaraldehyde in 100 mM Na cacodylate at pH 7.4 (sometimes with addition of 1% formaldehyde). Thorough washing in buffer of the specimens after fixation, osmication (2% osmium tetroxide in cacodylate buffer), contrasting with uranyl acetate (for 30 min in a saturated aqueous solution), dehydration with graded ethanols, infiltration with resin (Araldite) and curing at 60˚ C for 4–6 days were carried out as per standard procedure for transmission electron microscopy. Blocks were trimmed by hand, and semi-thin Sects. (1–3 µm thick) and thin sections (about 100 nm thick) were cut with glass knives. The semi-thin sections were examined unstained in a phase contrast microscope or after staining with toluidine blue. Thin sections were collected on copper grids, contrasted with uranyl acetate and lead citrate and examined in a transmission electron microscope.

### Planes of section

**Fig. 1:**
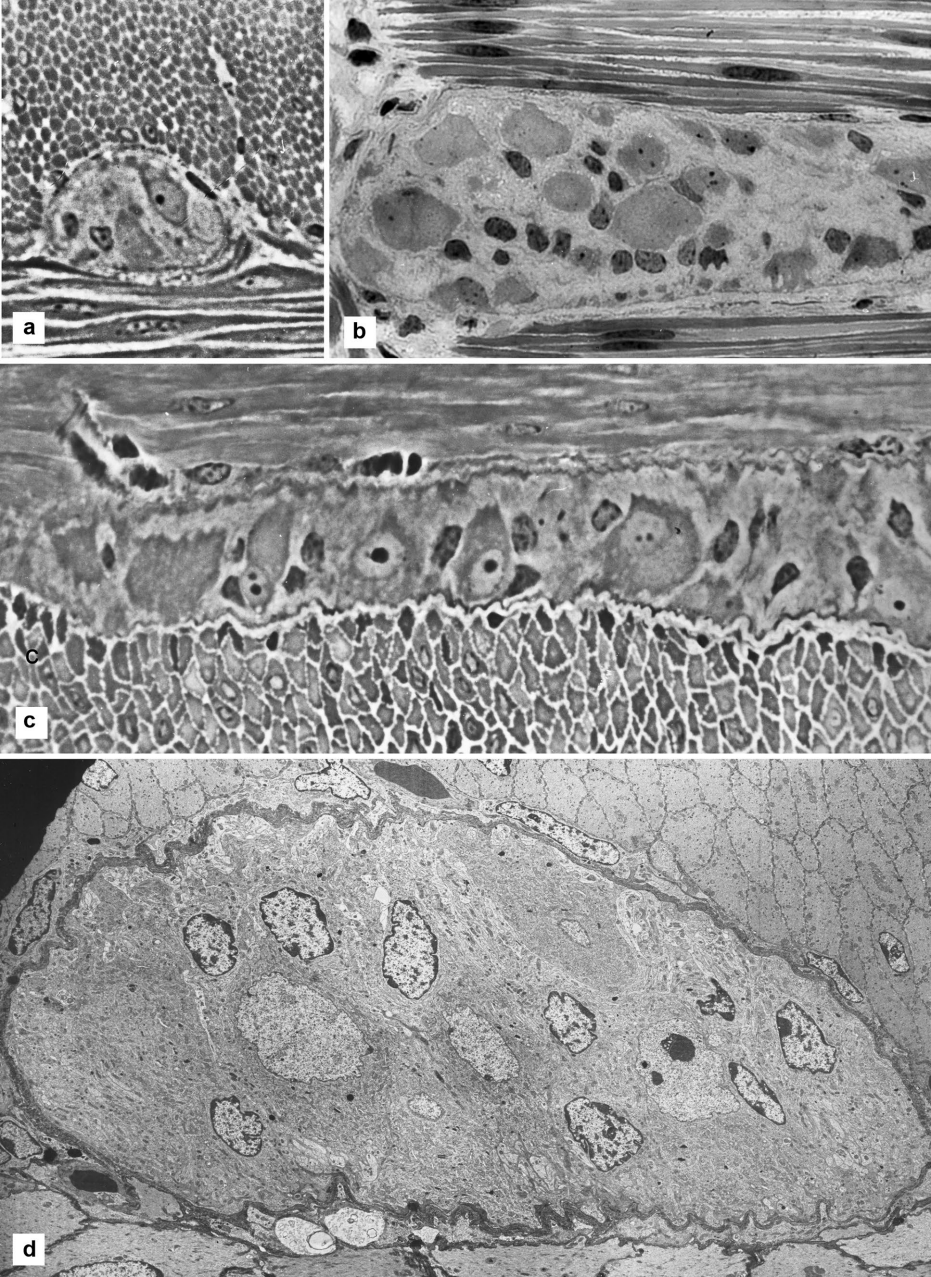
Overall views of Auerbach’s ganglia. **A** Light micrograph of a section across the wall of the intestine, transverse to the circular muscle (top) and to an Auerbach’s ganglion. The ganglion shows two nucleated nerve cells. Width of the microscopic field: 120 µm. **B** Light micrograph showing a ganglion on a section parallel to the serosa (tangential section). At top and bottom is the circular muscle cut lengthwise. Within the ganglion, glial cell nuclei are small dark roundish elements, while ganglion neurons, much larger, are grey and in smaller number than glial cells. Width of the microscopic field: 120 µm. **C** Light micrograph of a section transverse to the wall of the intestine transverse to the longitudinal muscle (bottom). An Auerbach’s ganglion is sectioned along its length and it shows several neurons, many with nucleus and nucleolus. More than a dozen glial cells show their nucleus as a small dark ovoid or triangular element. Width of the microscopic field: 250 µm. **D** Electron micrograph at low magnification of a ganglion lying between circular muscle (top) and longitudinal muscle (bottom). Nine glial cell nuclei (incrusted with dense material beneath their envelope) and some neural nuclei (the one to the right showing a nucleolus). The structure of the neuropil is not resolved at this magnification. Width of the ganglion 80 µm

The present data, especially those used for quantitation, were mostly from transverse sections of a ganglion, that is sections cut on a plane orthogonal to the thickness of the intestinal wall and transverse to the circular muscle. Some ganglia were cut in longitudinal section that is parallel to the circular muscle, and some were in tangential section that is parallel to the serosal surface (Fig. 1A, B, C).

The transverse sections offered the best views of ganglia and connecting strands, since all cell processes lie predominantly circumferentially to the gut wall that is parallel to the circular musculature, and are best seen and measured in transverse section.

### Montages

The bulk of the work was carried out on photographic montages covering the entire area of a ganglion or a connecting strand in a transverse section. Montages were prepared by assembling 5–30 electron micrographs at medium magnification (6000–12,000 ×) into a single image. The original micrographs were on either film or digital. Digital micrographs were assembled electronically. Micrographs on film were first printed (usually at 2.5 ×), then scanned (at 600 ppi) and assembled digitally. Scanning from the prints gave better results than direct scanning of the photographic negatives. Montages are laborious (compared with low magnification micrographs), but they preserve higher resolution to investigate cytological details.

### Morphometry

A digital version of each montage was made, either by scanning the prints on a flat-bed scanner or by using the ganglia from the digitally scanned negatives. These files were imported into FreeHand (version 10 or version MX, both of which work only on Apple obsolete operating systems, OS.X.6 or earlier). In FreeHand, it was possible to trace every cellular profile in a ganglion section (in some montages, the outlines of more than 2000 cell elements were traced). Each tracing was obtained by placing dots (mouse clicks) over the length of the membrane outlining a cellular element. The computer then constructs the Bezier curves for that series of dots and joins them into a single line (the perimeter of that particular element). With an attentive positioning and an optimal number of points, the lines have a good overlap on the element of the micrograph. It may be of interest that in all the curved lines that make up the profile of these processes, a mathematical treatment of the co-ordinates of just three points can create a curved line exactly superimposed on the curved membrane of the biological structure.

Contrast-enhancing, digitization and colour coding helped the human eye to define each component under study, its size, shape, position and relationships. The number of each type of element, their sectional area and the length of their outline (the membrane of the component) were obtained by means of the NIH software ImageJ (Fiji) in the public domain. In addition, all the mitochondria within a ganglion were traced, recording their number, size (that is, area and perimeter of their profiles in thin sections) and position.

The cellular profiles measured were to a very large extent transverse or near-transverse sections of the ganglion components. Because these elements are roughly fusiform, without a top or a bottom, the measures of sectional area reflect accurately the values for volume, and the length of the perimeter of the component is a valid indicator of the surface area of that component. All the cellular elements observed are, in fact, “profiles” of the elements, that is, they are the outline of that part of each of them that was caught in the histologic section. An “axon”, for example, is actually an axon profile (in many cases, this point is left implicit). Numerical data are presented with two decimal figures. This does not indicate the level of accuracy (usually limited to three significant digits), but it is used to identify measurements and calculated values. Percentage values are formatted with one decimal digit.

## Results

### General view of ganglia

**Fig. 2:**
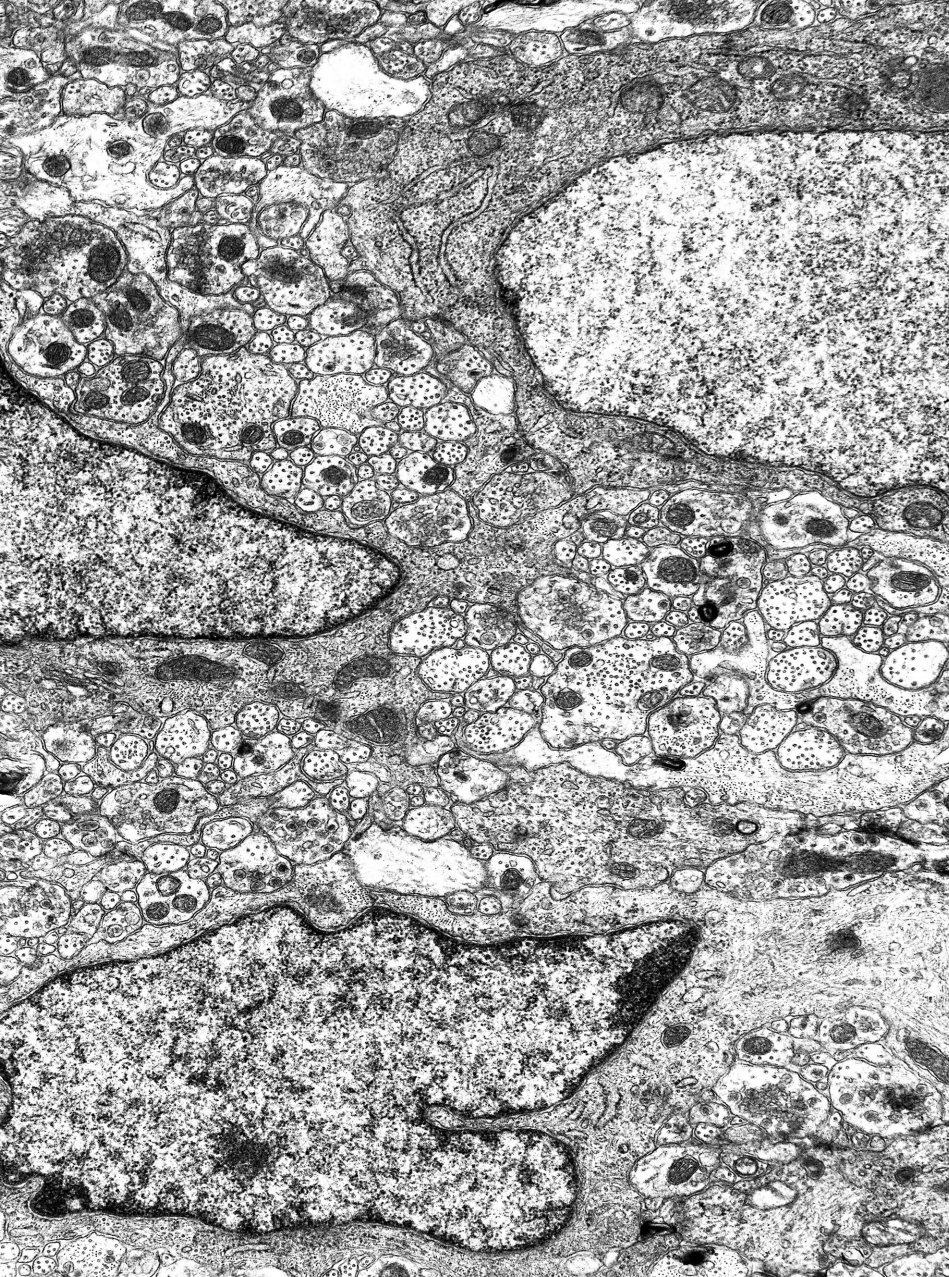
A panoramic view of the neuropil by electron microscopy. A nerve cell with nucleus is at top right corner; a nucleated glial cell is half-way along the left border, and another one is at the bottom. The section is transverse longitudinal, with the circular muscle side at the top. If a piece of tissue of this size could be cut again, orthogonally, it would produce more than 130 vertical sections. Width of the microscopic field: 12 µm

Large views of an Auerbach’s ganglion show the complexity, compactness and miniaturization of this tissue, a tight assembly of neurons and glial cells. By light microscopy, glial cells in these ganglia are readily identified via their nucleus. The nuclei are 2–3 µm in width, rather uniform in size, mostly ovoid in profile, not always smooth, densely rimmed and without a visible cytoplasm around them. In strong contrast, the neuronal nuclei are much larger (4–10 µm), optically lighter, close to round in profile and often displaying a nucleolus (Fig. 1). The orientation of the plane of section has a critical influence on the data. The arrangement of the ganglionic components shows up somewhat differently in transverse section (of the ileal wall) that are parallel to the circular muscle (Fig. 1A), or in sections parallel to the longitudinal muscle (Fig. 1C), or in sections parallel to the serosal surface (Fig. 1B). There is no apparent pattern in the distribution and position of glial cells and of neurons, in any of the section orientations. The compactness of the ganglia is immediately clear, with no evidence of spaces between the cells. These structural points are evident also in the electron micrographs, where even the smallest processes of neurons and glia are resolved at the appropriate magnification (Figs. 1D, 2).

It is well known that there is no connective tissue in the ganglia, and they are lined by a single, continuous basal lamina. Collagen and some fibroblasts lie directly around a ganglion; there is no connective tissue capsule, and no closeness or association of the ganglia with blood or lymphatic vessels. The musculature lies very close on all sides.

Beneath the basal lamina, the surface of the ganglion is a tessellated pattern contributed by glial processes (more than half of it), nerve cells (including an umbrella-like extension that some have), dendrites and axons. The outline of ganglia and strands is smoothly curved, without concavities and approaching the circular shape in the transverse profiles; it becomes slightly corrugated when the musculature is contracted.

Neuronal cell bodies occupy only part of the transverse section of a ganglion, in a variable proportion, ranging from more than half of the sectional area to close to zero. The remaining of the sectional area (the non-neuronal-cell-body part), known as neuropil, is equally variable in extent, in the reverse proportion; it is made up of the neuronal processes (dendrites and axons) and all the glial elements (cell bodies and processes).

#### Quantitative composition of ganglia

**Fig. 3:**
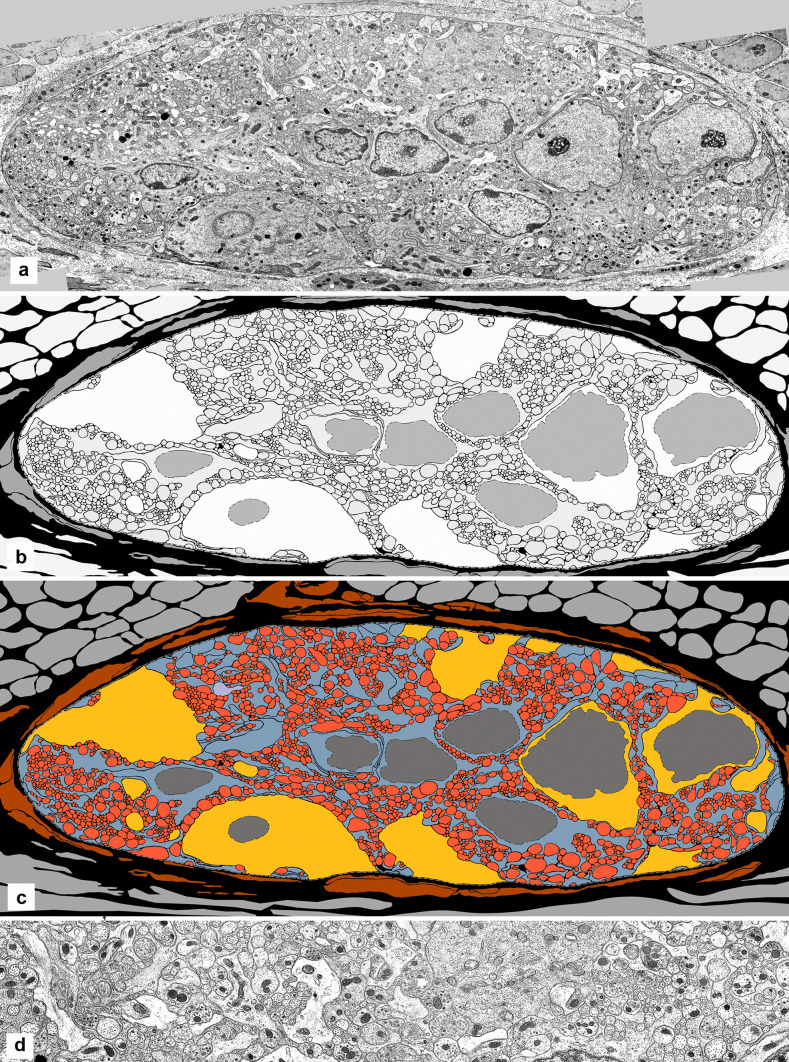
Auerbach’s ganglion and tracing derived from it for morphometry. **A** Electron micrograph of the full section through a ganglion (a montage), showing several nerve cell bodies (three of them nucleated) and a large expanse of neuropil. **B** In the same ganglion, all the cellular profiles (nerve cell bodies and their nuclei, dendrites, axons, glial cells and their nuclei, and glial processes) are traced digitally. **C** In the tracing, the axons are colour-coded in red, the neuronal cell bodies in yellow, the glia (cell bodies and processes) in blue and the nuclei in grey. Fibroblast processes around the ganglion are in brown, and the muscle cells in light grey. The extracellular space is in black. **D** Micrograph of an area of the same ganglion, at higher magnification, at the centre and top of the images above. At this magnification, the mitochondria are readily identified as black dots of fairly similar size. Width of the ganglion: 75 µm

The photographic montages of several ganglia were traced and subjected to quantitative analysis to establish numbers, sectional areas and membrane lengths of all their components (Table 1, and Fig. 3). The sectional areas of nerve cells, axons and dendrites, glial cells and glial processes, added together amount to about 95% of the sectional area of the whole ganglion. The difference (approximately 5%) is a rough measure of the optical gaps between the cellular elements (referred to as “extracellular space” or “intercellular space”, on the basis of its appearance in micrographs).

**Table 1:** Areas of ganglion elements. Seven ganglia were analysed (three-letter code in the third column), by measuring the sectional areas in micrometres as a percentage of the summation of the area of all the elements, and the lengths (or perimeter) of their plasma membrane. In the second column is the number of elements of the given type in that ganglion

		Code of ganglion	Sectional areas in µm^2^	Areas	Membrane lengths in µm	Length
				%		%
Ganglion	1	AFJ	700.1	100%	2436	100%
	1	AFK	1010.48	100%	5088.74	100%
	1	BHT	1051.75	100%	5393.6	100%
	1	BIJ	1763.21	100%	9774.49	100%
	1	BQA	624.46	100%	2871.22	100%
	1	BQB	315.8	100%	2594.24	100%
	1	BSN	1383.82	100%	5134.13	100%
Nerve cells	2	AFJ	312.3	44.60%	111.2	4.60%
	4	AFK	313.25	31.00%	212.01	4.20%
	5	BHT	348.16	33.10%	227	4.20%
	7	BIJ	736.95	41.80%	441.52	4.60%
	3	BQA	355.29	56.90%	171.03	5.90%
	0	BQB	–––-		–––-	–––
	6	BSN	581.23	42.00%	114.96	2.20%
Axons and dendrites	1196	AFJ	197.4	28.20%	1507	61.90%
	1658	AFK	317.39	31.40%	2393.49	47.00%
	2386	BHT	328.64	31.35	2769.43	51.40%
	2651	BIJ	509.25	28.90%	4881.01	49.90%
	1057	BQA	185.15	29.60%	1575.23	54.90%
	1187	BQB	198.78	62.90%	1607	61.90%
	2292	BSN	467.71	33.80%	2921.13	56.90%
Glial cells	3	AFJ	91.7	13.1	188.6	7.20%
	4	AFK	134.97	13.4	103.2	2.00%
	5	BHT	144.9	13.8	274.79	5.10%
	4	BIJ	155.91	8.8	387.21	3.90%
		BQA	–––	–––	–––	–––
	1	BQB	51.84	16.4	176.82	6.80%
	3	BSN	101.01	7.3	130.41	2.50%
Glial processes	167	AFJ	98.7	14.10%	629.2	25.80%
	682	AFK	244.87	24.20%	2380.04	46.80%
	438	BHT	230.05	21.90%	2122.38	39.40%
	630	BIJ	361.1	20.50%	4064.75	41.60%
	223	BQA	84.02	13.50%	1124.96	39.20%
	138	BQB	65.18	20.60%	810.42	31.20%
	642	BSN	233.87	16.90%	1967.63	38.30%
All neuronal		AFJ	509.7	72.80%	1618.2	66.40%
		AFK	630.64	62.40%	2605.5	51.20%
		BHT	676.8	64.60%	2996.43	55.60%
		BIJ	1246.2	70.70%	5322.53	54.50%
		BQA	540.44	86.60%	1746.26	60.80%
		BQB	198.78	62.90%	1607	61.90%
		BSN	1048.94	75.80%	3036.09	59.10%
All Glial		AFJ	190.4	27.20%	817.8	33.60%
		AFK	379.84	37.60%	2483.24	48.80%
		BHT	374.95	35.70%	2397.17	44.40%
		BIJ	517.01	20.30%	4451.96	45.60%
		BQA	84.02	13.50%	1124.76	39.20%
		BQB	117.02	37.10%	987.24	38.10%
		BSN	334.88	24.20%	2098.04	40.90%

The quantitative data, such as those presented in Table 1 obtained from full sections of 7 ganglia, are rather variable.

However, when expressed as percentage values, they present consistent patterns in all the ganglia (with only few measurements out of line) (Table 2).

**Table 2:** Extent of the neuropil

	Code of ganglion	Sectional areas in µm^2^	Areas	Membrane lengths in µm	Length
			%		%
Neuropil	AFJ	387.8	100%	2324.6	100%
	AFK	697.23	100%	4876.73	100%
	BHT	703.59	100%	5166.6	100%
	BIJ	1026.26	100%	9332.97	100%
	BQA	269.17	100%	2699.99	100%
	BQB	315.8	100%	2594.24	100%
	BSN	802.59	100%	5019.17	100%
Neural part	AFJ	197.4	60.90%	1507	64.80%
	AFK	317.39	45.50%	2393.49	49.10%
	BHT	328.64	46.70%	2769.42	53.60%
	BIJ	509.25	49.60%	4881.01	52.30%
	BQA	185.15	68.80%	1575.23	58.30%
	BQB	198.78	62.90%	1607	61.00%
	BSN	467.71	58.80%	2921.13	58.20%
Glial part	AFJ	190.4	49.10%	817.8	35.20%
	AFK	379.84	54.50%	2483.73	50.90%
	BHT	374.95	53.30%	2397.17	46.40%
	BIJ	517.01	50.40%	4451.96	47.70%
	BQA	84.02	31.20%	1124	41.70%
	BQB	117.02	37.10%	987.24	38.10%
	BSN	334.88	41.70%	2098.04	41.80%

The neuronal cell bodies are the largest component of the ganglion, even with their variable presence in different histologic sections, and as evident in the tables. Averaging all the ganglia, the nerve cell bodies take up almost 40% of the space, axons and dendrites slightly more than 30% and all the glia slightly less than 30%.

As regards the length of the cell membrane (or plasmalemma) of all the elements (that is, their perimeter, which is a measure of their external surface area), the contribution of nerve cell bodies (roughly 5%) is small, due to their large volume, ovoid shape and relatively smooth outline. Cell membranes of axons and dendrites are about 50–60% of the total of the ganglion and those of all glial elements about 35–45%.

By leaving out the nerve cell bodies and focusing on the neuropil (which is also the entirety of the connecting strands), the total neuronal area exceeds the total glial area by 55–65% to 45–35% (Table 2). These are large values compared to the 5% provided by the nerve cell bodies (but they include the amount provided by the dendrites).

Overall, the extent of cell membrane, even when the nerve cell bodies are included, is some fifty times the outer surface of the ganglion (and more in the larger ganglia).

#### Compactness of the assembled elements

The gap between adjacent elements (or, rather, the electron-lucent space between them), whether glial-glial, glial-neuronal or neuronal-neuronal, has a fairly regular width of about 15 nm; it is larger in the triangular areas where three elements are close together. The width between adjacent membranes is never further reduced, except at the rare gap junctions between glial elements.

Most of the glial membrane is apposed to neural elements, and glial-glial appositions are less common. Some glia covers a large area of a nerve cell body and here the covering is generally smooth but very rarely complete. The majority of neurons have a region of their surface that is uncovered by glia and lies directly under the basal lamina.

#### Size and shape of glial cells

The size range of glial cells could not be determined, but these cells are small and form a relatively uniform population in this respect, compared with the nerve cells, which are remarkable for their wide size range. Glial cells do not exceed in size the smallest ganglion neuron.

The cells are slim, often just a rim of cytoplasm of 2–3 µm around the nucleus. Almost invariably, an area of the nuclear surface lies within a fraction of a micrometre from the cell membrane. The limits of a cell body can be indistinct, because of the innumerable processes emerging from it and the continuous indentations from adjacent elements. Some large glial expansions may cover part of the surface of a nerve cell.

More commonly, the emerging large processes of glial cells are laminar (rather than cylindrical), and they are visualized as extending radially when seen in transverse sections of the ganglion.

Usually, the radial laminar processes extend from the cell body for 20–40 µm in all directions among the axons. The laminae divide repeatedly, diminishing irregularly in thickness with the distance from the cell body; occasionally they present expansions, but they never merge with each other. Their numerous branching points are curved, often with a semi-circular pattern, hence with great divergence. Some laminar processes reach the ganglion surface (not uncommonly, both sides of it, that is, towards the circular and the longitudinal muscle) where they generally expand into flat terminals beneath the basal lamina (see further down).

The smallest laminae tend to break into minute finger-like terminations that can measure as little as 60 nm in width (the cytoplasmic space between opposed membrane can be reduced to just under 30 nm, but not beyond). As in the cell bodies, the surface of glial processes is continuously indented by the surrounding neural processes. In consequence, more than 9/10 of the glial surface is concave (curved inwards), expanding the amount of membrane and reducing the amount of cytoplasm. The opposite is the case with axons: Their profile is close to circular, so that the amount of membrane (axolemma) is maximally reduced for the amount of cytoplasm (axoplasm). Overall, the glial processes are shaped by the surrounding neuronal elements, whereas glial processes are never seen to affect the shape of a neuronal element.

Rough calculations of the surface to volume ratio confirm this point. Neuronal processes (predominantly close to circular in profile in the 12,427 that were traced) have a total sectional area of 4900 µm^2^ and a total membrane length of 18,900 µm, while the glial processes (2920 of them) had a total sectional area of 2000 µm^2^ and a membrane length of 14,000 µm. The volume to surface ratio is calculated as 1 to 3.9 for the axons and 1 to 7 for the glia, that is, in glia there are some 7 square micrometre of membrane for every cubic micrometre of cytoplasm, as opposed to about 4 to 1 in axons.

#### Glial cell membrane

The cell membrane has a uniform appearance in glial cell bodies and processes. Its electron-dense (visible) part, separated by a lucent gap of 15 nm from the membrane of adjacent processes, is also separated, on the cytoplasmic side, from the cell organelles by an electron-lucent space of 10–15 nm. This space is never penetrated by cell organelles, such as microtubule or mitochondria.

A small number of poorly developed junctions of the adherens type occur between glia and neurons (but not between two glial elements) and a small number of gap junctions are found between glial elements (but not between a glial and a neuronal element). Presence of gap junctions between glial cells agrees with the die coupling observed in these cells (Hanani et al. 1989). Gap junctions are rare enough to make quantitative estimates too difficult; the reliable evidence of these junctions is based on preparations by freeze-fracture, illustrated elsewhere (Gabella 1981).

In contrast, at the ganglion surface, the cell membrane of every glial element in contact with the basal lamina (in the ganglia but not in the connecting strands) has conspicuous condensations of electron-dense material on its cytoplasmic side.

All around the ganglion, large bundles of gliofilaments are inserted into these areas, referred to as the basal lamina domain of the cell membrane of glia. There is no equivalent inside the ganglion, where only few filaments are inserted on the membrane. Gliofilaments (the main component of the cytoskeleton) are abundant in glial cell bodies and in large processes. However, gliofilaments are absent in large areas of some glial cells, and they also do not extend into the small glial processes (which would then not be visible by GFAP histochemistry). While thick and long bundles of gliofilaments are common, the predominant arrangement is in small bundles that crisscross each other in several directions.

#### Axonal contacts with glia

**Fig. 4:**
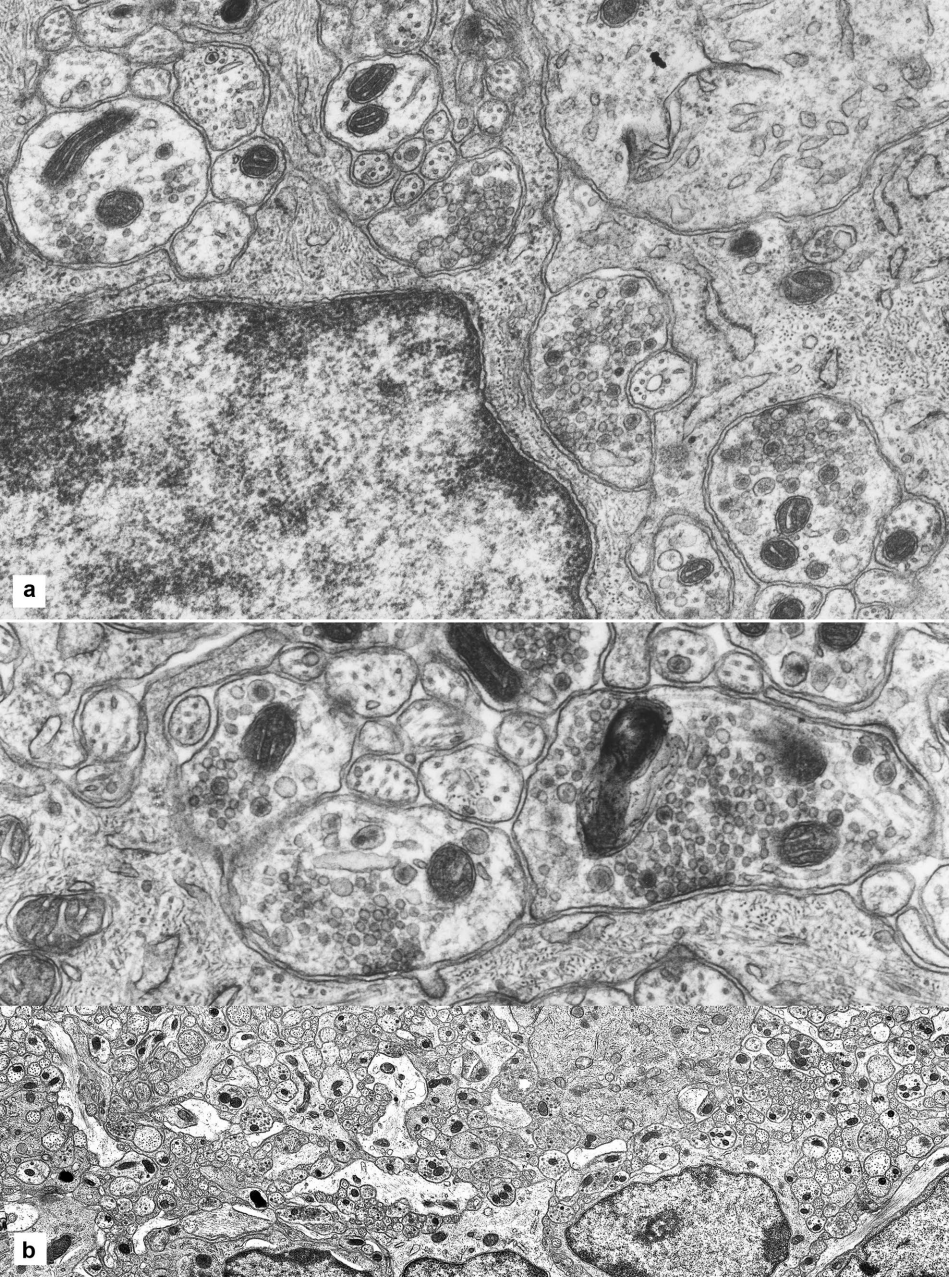
Axon-glia contacts. **A** A glial cell, with its nucleus, occupying the bottom left quarter of the picture, and one of its processes at centre have two large vesicle-studded axons abutting on them (circled). Another axon-glial contact is visible on the glial cell at the right (circled) and one at the far left (circled). The four axons (nerve endings or varicosities) show vesicles aggregating against a membrane density; no specialized structures appear on the glial side of these contacts. Width of the microscopic field: 9 µm. **B** A long glial process issuing from a cell body, to the left, is contacted by two large nerve endings (varicosities) mainly occupied by mitochondria and vesicles; the latter are clustered under a membrane density that is visually identical to those found in synapses. No structural specializations are visible on the glial side. Width of the microscopic field: 6.5 µm

Contacts between vesicle-containing axons (usually varicosities) and glia are abundant. In several of these, in the glial cells of the ganglia, vesicles are clustered on an area of the axonal membrane which is encrusted with some dense material (Fig. 4A, B). However, no structural specialization is noticeable on the corresponding membrane area of the glial cell or process. The space between the two elements at these points of contact is flat or minimally curved. No other specializations are noted at these sites on the glial side, or any pattern in their distribution over the glia.

These axonal-glial contacts are frequent in the neuropil. It is rare to look at a microscopic field, say an area of 20 × 30 µm, without finding at least one. Synapses (axo-somatic and axon-dendritic) are noticeably less frequent.

### Glia-axon spatial relations

**Fig. 5:**
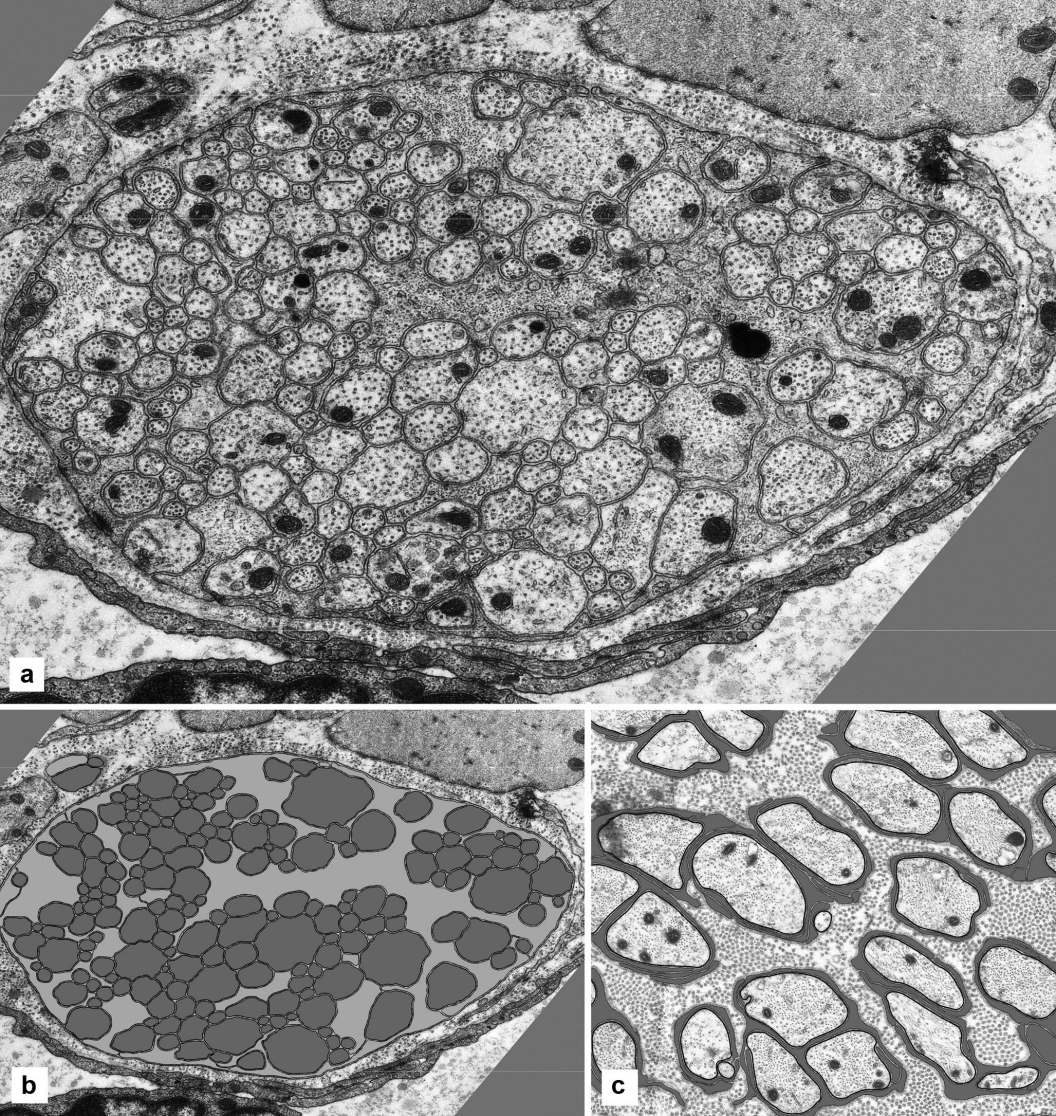
Glial processes and axons. **A** A connecting strand in transverse section comprises glial processes and axons in a compact assembly. The glia is in the form of irregular laminae that make extensive contacts with the axons, separating them in groups, and that reach the surface of the strand. However, the majority of the axons are not in contact with glia but only with each other. Width of the microscopic field: 12 µm. **B** The same connecting strand with all the neural and glial processes digitally traced. The neural elements are filled in grey, the glial components in light grey. The black line is the tracing of the basal lamina. **C** Transverse section of a large intramural branch of the pelvic nerve in the wall of the urinary bladder, with axons and glia digitally traced. The glial processes are filled in grey. Every axon is fully sheathed by glia. Width of the microscopic field: 9 µm

All glial processes, large or small, lie in contact with a neural element. Glia-to-glia contacts are less common. Many axons, but far from all, are in contact with a glial element, shallowly or deeply indenting the surface of the glia. Many axons, however, are not in contact with glia (at the level of a single transverse section), let alone wrapped all around by glia (Fig. 5A, B). This is in strong contrast with all other autonomic nerves (non-enteric), where the glia forms a complete sheath around every axon and every neuron (Fig. 5C).

#### Mitochondria

**Fig. 6:**
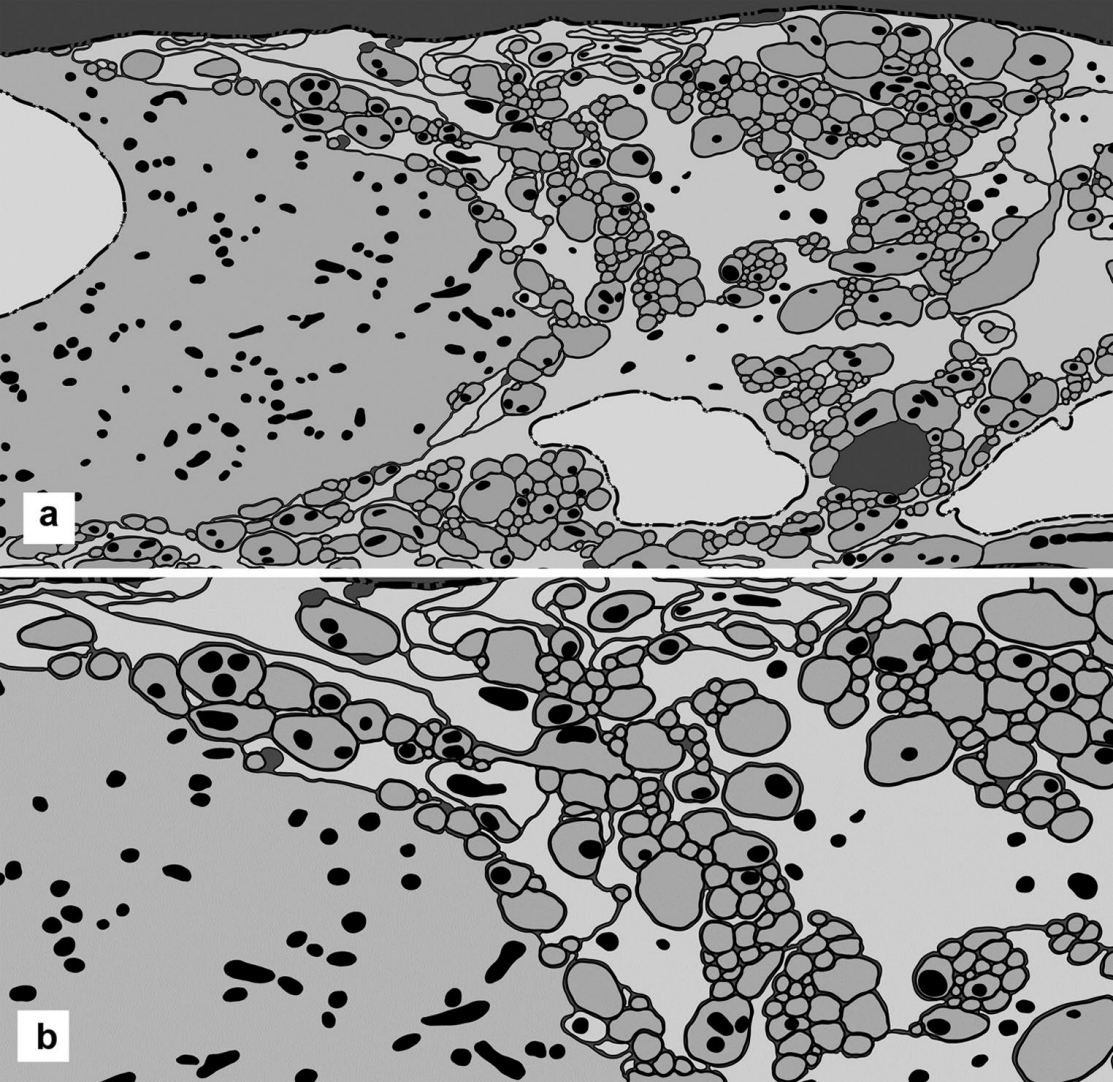
Distribution of mitochondria. **A** In a ganglion digitally traced (as in Fig. 3), a nerve cell is at the left and two glial cells at bottom right. Three nuclei are in white, and the neuronal cytoplasm and all the neural processes are in light grey; glial cells and glial processes are in dark grey. All the mitochondria are traced and filled in black. The background is also in black, filling all the extracellular space (hardly existing within the ganglion). Width of the microscopic field: 13 µm. **B** In the same preparation, the area at top centre is shown at higher magnification. Width of the microscopic field: 9 µm

The traced and digitized montages provide a reliable way to count the mitochondria, measure their size and note their location in every component of a ganglion (Fig. 6A, B). Here, the extent of the chondroma is expressed as percentage of the sectional area of cytoplasm of all processes and cells (excluding the area of the nuclei) that is occupied by mitochondria (Table 3, with the data from 5 ganglia).

**Table 3:** Extent of mitochondria in 5 ganglia

GP ileum myenteric	Code of ganglion	Cellular areas in µm^2^	Mitochondria		
			number	area in µm^2^	% area
All ganglion elements (minus nuclei)	AFJ	582.6	603	30.19	5.20%
	BIJ	1398.1	1681	104.39	7.50%
	BQA	523.6	613	44.41	8.50%
	BQB	293.69	388	18.16	6.20%
	BSN	1201.8	1027	76.06	6.30%
Nerve cells (minus nuclei)	AFJ	231.8	249	14.69	6.30%
	BIJ	430.5	495	37.85	8.80%
	BQA	254.43	293	26.74	10.50%
	BQB	––––	–––	–––	–––
	BSN	454.52	448	42.88	9.40%
Axons and dendrites	AFJ	197.4	275	µ	6.60%
	BIJ	509.25	908	12.93	9.40%
	BQA	185.15	268	47.85	7.80%
	BQB	198.78	321	14.35	7.00%
	BSN	467.71	458	13.93	6.10%
				28.46	
Glial cells (minus nuclei)	AFJ	54.7	28	1.29	2.40%
	BIJ	97.25	52	3.71	3.80%
	BQA	––––	––	–––	–––
	BQB	29.73	26	1.81	6.10%
	BSN	45.73	24	2.13	4.70%
Glial processes	AFJ	98.7	51	1.28	1.30%
	BIJ	361.1	226	14.98	4.10%
	BQA	84.02	52	3.32	4.00%
	BQB	65.18	41	2.42	3.70%
	BSN	233.87	97	6.24	2.70%

The total content (spatial density) of mitochondria varies in different ganglia, roughly between 5 and 8%, without indications of the basis of this variability. There is also a large variability in the mitochondrial density in the nerve cell bodies, roughly from 6 to 10% of the cytoplasm. Variability, although less extensive, is found also in the other components, namely, axons and dendrites, glial cell cytoplasm and glial processes (Table 3).

The most consistent data are in the ratios of the mitochondrial density in neural cytoplasm and in glial cytoplasm. Mitochondrial density in neurons is always in excess to that of glial, in some cases by a factor of 2.

## Discussion

### Technical consideration

The comparison of histologic sections of ganglia (1–2 µm thick) viewed by light microscopy and Sects. (0.1 µm) seen by electron microscopy illustrates the advance provided by the latter method. Associated technical limitations have been discussed elsewhere and they are only mentioned here. First, distortions occurring with the chemical fixation, with resin embedding and with sectioning are hard to control and to assess, and they do not affect uniformly all parts of a tissue. Second, extrapolation of the shape of a microscopic body from thin sections through it has limitations. Third, optical and electron densities in microscopic images are only aspects of the material density of the tissue. Fourth, there is an issue with sampling, since even in the best of these experiments the number of samples is relatively small. In a large ganglion, a complete transverse section, a tenth of a micrometre thick, may amount to less than one millionth of the ganglion volume. Therefore, the observations must be read minding the level of reliability that individually each of them may have, even when numerically expressed.

### Compactness and possible adhesion

The fine structure of the enteric glia in the electron microscope at the resolution of cell membrane and organelles appears highly variable and complex, even when only static views are provided and only at one point in time in the life history of the subjects (young adult guinea pigs). Variability is partly accounted for by the technique and the sampling, but it may also be related to the individuality of each subject and their life history, if one assumes that there is an element of “learning” in the processes of morphogenesis that produce a ganglion. With a visual approach, an immediately noticeable and constant feature is the compactness of the tissue, and the intimate interpenetration of glial and neuronal elements. To examine neurons and glia separately may be expedient but it enforces an artificial separation.

Glial and neural elements, which are in general easily distinguished visually, could not be more extensively mixed with each other than they are in these ganglia; mixed, and, presumably, functionally interconnected. An electron-lucent space of about 15 nm exists between the visible part of the cell membrane of adjacent elements (widened into a triangular area at the point where three elements are adjoined). Small variations in width can be accounted for by the embedding procedure and by slight changes in the orientation of membranes with respect to the plane of section; they do not invalidate the notion of a membrane-to-membrane separation of constant width.

The compactness is not achieved by external forces compressing the ganglion, and there is no capsule of any sort around ganglia and connecting strands. In addition, ganglia and strands always have a nearly-circular profile (in transverse sections), thus keeping the outer surface area of ganglia and strands to a minimum.

Therefore, the compactness (an observation) is generated intrinsically, and may (a speculation) be achieved by internal adhesion, that is, it may be due to adhesiveness of the surface of all cellular elements, which brings them together and keeps them together. Presumably, this adhesion is not cell-type specific, since all the elements are intermixed and bundled together.

However, it may vary in strength between components or may have some specificity, so that certain contacts are more likely to be formed or to be stronger than other. The adhesion is not strong enough to affect significantly the near-circular shape of many neuronal profiles, leaving a slightly wider area at the corners when three elements come together.

The 15-nm separation is not, strictly speaking, an extracellular space, because it must be occupied by rigid molecular elements that impede a closer apposition of the adjacent membranes, while at the same time—it is suggested—holding them together, as part of the adhesion mentioned above. It can be argued that the regular separation is maintained by an array of molecules working both as spacers and as holders of the adjacent surfaces together.

### Extent of glia

As regards space occupancy, in an early light microscope study, the nerve cells were shown to take up 35% of the volume of Auerbach’s ganglia in the ileum of guinea pigs (with marked differences in other ganglia and other animal species) (Gabella 1981). Here, by electron microscopy and on a smaller sample (27 nerve cell bodies in Table 1), the average value of the volume occupied by nerve cells is 38.6%, and almost 2/3 of the ganglion volume is neuropil. Of the neuropil, slightly more than half in volume is neuronal and slightly less than half is glial. The figures from a set of ganglia suggest a certain consistency in the extent of the glial and neuronal components, as if there were an optimal quantitative relation and some variation around it.

The extent of the glial component is substantial. However, it remains uncertain how the enteric glia compares in this respect with the glia in other districts of the nervous system. There are no data for making numerical comparisons. However, the impression is clearly that in other autonomic ganglia the ratio of glia to neurons is much higher than in the enteric ganglia. In the CNS, it is said that 60% of the cells are glial (in mouse) or even 90% (in humans) (Allen and Barres 2009). It is possible that the enteric glia, even when making up one-third of the ganglion volume, and prominent as it appears in these micrographs, is not as highly developed as the glia of other areas of the nervous system. In fact, visually the contrast in the extent of the glial is outstanding. In sympathetic and parasympathetic ganglia, glial cells form a full coat around the nerve cells (except for the synapses and the emerging dendrites). In contrast, in Auerbach’s ganglia, only rarely do glial cells enwrap completely a ganglion neuron. Furthermore, in other ganglia (and in nerves), every axon is fully sheathed by a glial process (with its mesaxon), whereas this is far from the case in the enteric plexuses. In a single histologic section of a ganglion, many axons are not even in contact with a glial process. Nevertheless, the arrangement of the glial processes is such that they spread as far away as possible from the cell body as if to maximize contact with neural elements.

The developmental reason of this neural-glial relationship is not known; one can suppose that in the Auerbach’s plexus, there are not enough glial cells to provide the full covering of neural elements, an arrangement substantially different from the one found in other autonomic ganglia outside the gastro-intestinal tract.

### Cell membrane of glia

Inside the ganglia, the uniformity of appearance of the cell membrane of glia has been noted. The scarcity of cell junctions of the adherens type, their poor development and their limited association with gliofilaments are remarkable features, and they may have mechanical significance. All around a ganglion, a distinct domain of the cell membrane of glia is associated with the basal lamina and bears a massive insertion of gliofilaments. The adhesion of cell membrane and basal lamina is critical and specific for the development of this domain.

The source of this basal lamina is unknown, or which cells make its molecular components (possibly the glial cells themselves).

Once assembled, the basal lamina has full continuity over the various cell elements that appear at the ganglion surface. The lamina probably imparts mechanical strength to the outermost part of the ganglion, making it rigid and only moderately pliable or extensible (a sort of semi-solid material). The other glial membrane domain, far more extensive and all inside the ganglion with few junctions and a limited insertion of gliofilaments would be highly pliable and deformable (a sort of semi-fluid material).

### Glial cell shape

Glial cell bodies are small (compared with neurons), relatively uniform (in sharp contrast with neurons) and with an irregular outline indented all around by the adjacent neural processes. Their shape is passive, in the sense that there is no contractile ability or any active movement in this glia, and it is imparted by the surrounding neural elements (while glia never influences the shape of axons or of nerve cells). The three-dimensional architecture of the glial cytoskeleton (mainly the gliofilaments) must greatly influence the shape passively taken by the glia from stresses imposed on it.

An impressionistic account of the shape taken by the components of the neuropil could be that in the glia, the growth of its cell membrane is maximized, or at the expense of its cytoplasm. In contrast, in the axons, the growth of the axolemma is kept to a minimum for the given amount of axoplasm. In addition, the penetration of glial processes among the axons, while they extend away from the cell body, suggests an affinity of glial cell membrane for axolemma.

### Axon-glial contacts

Specialized contacts between nerve ending (strictly speaking, the varicosities located along the length of terminal axons) and glia are found throughout the ganglion. They have been noted before (Gabella 1981) but have not attracted attention and remain puzzling. However, several studies have explored the functional influence of neurons on enteric glial cells (e.g. Seguella and Gulbransen 2021; Delvalle et al. 2018), along lines to which the present data do not contribute.

Because no structural specialization is visible on the glial side, they are not regarded as cell-to-cell junctions (or as possible synapses), but simply as contacts. They are identifiable unequivocally, they are numerous (more common that the synapses) and they are characterized, on the axonal side, by the abundance of vesicles, some of which are clustered against a patch of the membrane that is encrusted with dense material.

A suggestion compatible with the data is that axonal vesicles, which are predominantly grouped in clusters all along the axons, at some sites or at some times make contact with the axonal membrane, producing arrangements highly specialized, irrespective of what is the other element of the contact. It will be hard to find out what happens at these specific sites, and whether they serve a role in ganglion physiology, motor or sensory. For the moment, the possibility that neurotransmitters are released at those sites cannot be dismissed and it even seems likely.

Then, should it be the case that transmitters are released at non-synaptic sites, it would imply that the circuit activities of the ganglia are not exclusively by synaptic transmission; therefore, additional mechanisms would be playing a role in the local nerve activity, including the afferent processes.

### Mitochondria

The morphometric method employed here on mitochondria is accurate for quantitative analysis (with fewer sources of error than biochemical methods), and the figures obtained are regarded as fairly reliable.

The total content of mitochondria is 5–8% of the entire cytoplasm in a ganglion, a relatively high value compared with other tissues, for example the adjacent musculature. There is some variation in mitochondrial density between individuals, which is genuine but for which there is no explanation. There is also some variation between nerve cells of a single ganglion that is not discussed here, but which suggests functional differences within the neuronal population.

As to the glia, the spatial density of mitochondria (in number of units and in percentage volume) is markedly and consistently smaller in glia than in the neural elements. The difference is highly significant, and in some cases the mitochondrial density in glia is only about half than that of neurons. Overall, about a quarter of the total of mitochondria is in the glia, and three quarters in the nerve cells and processes.

The number (or volume) of mitochondria is not a measure of the actual metabolic activity of a ganglion, but rather is an indication of the maximal metabolic (aerobic) activity that can be produced at the various sites. In this case, the metabolic activity of the glia (as a whole in the ganglion or as per volume of cytoplasm) is significantly lower than that of the neural cytoplasm.

The significance of this variance is not yet clear, but it indicates a large difference in the metabolic activity of the two components of the ganglion, with a substantially smaller potential in the glial one. This observation does not fit with the glia’s proposed role of metabolic support to the neurons.

### Roles of glia

Little is known about the functions served by the glia in the enteric ganglia. Actually, a wide range of roles have been proposed from various laboratories, role that involves especially the cells located in the mucosa. Overviews of this aspect of the field are available in the literature (see the “Introduction” section), and the present descriptive observations make no contribution to the proposals most explored at this time.

The extent of the glia in the Auerbach’s ganglia and the intimate relationship with the neurons indicate the existence of essential roles, in physiology and in development (hence in pathology), but it also makes in situ investigations very difficult. Besides, the ganglia are both under intense stress and subject to extensive changes in shape. Given the extreme (and, in a broad sense, reversible) deformability of the intestinal wall and its ganglia, a mechanical role for the glia comes readily to mind.

Such a role is usually overlooked as a “not only” one, but how it is played out and regulated is far from clear, when their essential role is regarded as that of nourishing neurons and maintaining homeostasis (see the “Introduction” section). A mechanical role of glia in the CNS, if it exists, would be substantially different.

The dimensions of a ganglion can change markedly (by imposed stress from the muscle, that is passively). There is much displacement and re-shaping of its elements and each of its axes easily doubles or halves when the muscle layers contract. The large deformation of the ganglia, the absence of a proper extracellular space (or of some kind of empty space to facilitate the movement of parts) and the constancy of the volume of cells (reduced to sealed containers of incompressible fluid) are three challenging aspects of the mechanics of this material.

A factor worth mentioning is that under mechanical stress, the axons can be displaced but they are almost non-deformable, given their shape and surface to volume ratio. Therefore, most of the form change within a ganglion, as caused by muscle contraction, involves the glia. The laminar, rather than cylindrical, shape of glial processes should permit the axons to be displaced in ganglia under compression without change in their size.

### Neurons and glia

The functional relationship between ganglionic neurons and glia must be very intimate and complex, as the microscopic views suggest. What exactly these reciprocal effects are is very little known, and there is need of additional work, unbiased by firm hypotheses. The quantitative data presented here imply a regulation of the extent, or volume, of the two elements (either reciprocally, or by an overriding control mechanism). The numerical ratio of glia to nerve cells also point to a regulation, and even more so do the differences in various ganglia, for example, with the extent of the neuropil and the number of glial cells per neurons being about double in ganglia of sheep than in mice (Gabella and Trigg 1983). It is probably significant that the extent of the glia is somewhat correlated with the average size of the ganglion neurons, as was observed in mammalian species of different body size.

On a wider perspective, the present observations may point to some scientific merit in descriptive accounts of complex structures, and to the value of looking at tissues in situ. Visual data, even if strictly episodic (that is based on observations of single cases), can add to our perception of biologic presences.

An interest in these materials is also in the morphogenetic processes that bring about certain structural arrangements and maintain them. The ganglia, minute, compact, well-outlined, self-contained are an attractive example of a structure originating by a process of self-assembly, even if it is still a rather mysterious one.
